# Sequence-specific processing of telomeric 3' overhangs by the Werner syndrome protein exonuclease activity

**DOI:** 10.18632/aging.100032

**Published:** 2009-03-17

**Authors:** Baomin Li, Sita Reddy, Lucio Comai

**Affiliations:** ^1^Department of Molecular Microbiology and Immunology, Institute for Genetic Medicine, University of Southern California, Los Angeles, CA 90033, USA; ^2^Department of Biochemistry and Molecular Biology, Institute for Genetic Medicine, University of Southern California, Los Angeles, CA 90033, USA

**Keywords:** Werner syndrome protein, telomere, exonuclease, aging, RecQ helicases

## Abstract

Werner syndrome is a premature aging disease caused by loss of function mutations in the Werner syndrome protein (WRN) gene.  WRN is a RecQ helicase that in contrast to every other member of this family of proteins possesses an exonuclease activity. The findings that cells lacking WRN activity display accelerated telomere shortening and WRN can be detected at chromosome ends suggest that this protein participates in some aspects of telomere metabolism. In this study we examined the impact of WRN on telomeric substrates with a 3' single-stranded overhang *in vitro* and show that WRN has sequence-specific exonuclease activity that removes several nucleotides inward with a periodical pattern from the 3' end of the telomeric overhang. This activity is strictly dependent on the presence of telomeric sequences in both the duplex DNA and 3' overhang DNA segment and is strongly inhibited by the telomeric factor POT1 but not TRF2.  These data demonstrate that WRN processes telomeric DNA substrates with a 3' single-stranded overhang with high specificity and suggest that this protein could influence the configuration of telomere ends prior to the formation of a protective t-loop structure.

## Introduction

Werner Syndrome (WS) is an autosomal recessive segmental aging disorder associated with a marked predisposition to cancer and vascular disease [1,2].  The first signs of this disorder appear after puberty and the disease is usually diagnosed in individuals 20 to 30 years of age. WS patients show increased predisposition to diseases observed during normal aging such as arteriosclerosis, osteoporosis, type II diabetes mellitus and a variety of tumors, primarily of mesenchymal origin [1,3]. Myocardial infarction (MI) and cancer are the most common causes of death among WS patients, with a median age of death of approximately 47  years.

WS is caused by mutations in the gene that encodes for the Werner syndrome protein (WRN), a protein that belongs to the class of enzymes termed RecQ helicases [4,5].  In contrast to other members of this family of helicases, WRN has an exonuclease domain, which is highly homologous to the nuclease domain of *E. coli* DNA polymerase I and ribonuclease D (RNase D) [6].  Helicase and exonuclease activities with a 3' to 5' directionality have been demonstrated *in vitro *using recombinant WRN [7-14]. WRN can unwind and/or hydrolyze a number of different DNA structures, from linear duplex DNA to single stranded regions of flap DNA substrates to synthetic replication forks and Holliday junctions [15,16].  WRN exonuclease is highly processive on substrates with a recessed 3' end structure, an activity that is independent of 5' overhang length and nucleotide sequence.  In addition, WRN displays weak length-dependent degradation of 3' overhang DNA substrates, which is independent of nucleotide sequence [17].

The presence of helicase and exonuclease activities have suggested that WRN may function in the processing of pathological DNA ends during DNA repair and/or recombination [18].  Moreover, since a subpopulation of WRN is localized at telomeres, it has also been proposed that natural DNA ends are genuine substrates of this enzyme [19,20]. Indeed, WRN has been shown to bind TRF2 and POT1 [21-23], two telomere-specific proteins, and *in vitro* studies have indicated that both TRF2 and POT1 stimulate WRN helicase activity on substrates that mimic telomere ends [20,22]. However, while TRF2 has been reported to either stimulate or repress WRN exonuclease activity [20,21], POT1 does not appear to influence this activity, at least in the context of the substrates tested in these studies.  Significantly, recent studies have indicated that WRN is important for maintaining the G-rich lagging strand of telomeric DNA [19,20] and our own work has demonstrated that WRN is required for proper telomere homeostasis by preventing the formation of extrachromosomal telomeric circles [24].

Human telomeres are composed of several kilobases of the repetitive hexamer TTAGGG and contain a 3' single-stranded DNA extension that is thought to loop back and invade the proximal complementary strand thereby leading to the establishment of a protective structure termed telomeric-loop (t-loop)[25-27]. The 3' overhang is generated either by removal of the RNA primer from the newly replicated lagging strand or by nucleolytic attach in the 5' to 3' direction after replication of the leading strand. In telomerase-positive cells, the 3' overhang is utilized as template by the RNA subunit of telomerase to extend telomeres.

Telomere homeostasis is maintained by a multiprotein complex that includes telomere-specific proteins including telomere repeat factors 1 and 2 (TRF1 and TRF2), which binds telomeres through a myb-like DNA binding domain located at the carboxyl-terminal end [28-30], and protection of telomeres (POT1), a G-strand specific single-stranded DNA binding protein [31]. These proteins are though to protect telomeres from end-to-end fusion and intra- or inter-telomeric recombination events [32,33]. Disruption of this protective structure activates a DNA damage response pathway and leads to cell cycle arrest, cell senescence or apoptosis [34-37].

To gain mechanistic insights on the function of WRN at telomeres, we have carried out exonuclease assays utilizing telomeric templates bearing a 3' G-rich overhang. Interestingly, we found that the 3' overhang of telomeric but not non-telomeric DNA substrates is specifically hydrolyzed by WRN exonuclease *in vitro*. 3' to 5' processing of the telomeric DNA substrates by WRN exonuclease activity is limited to nucleotides within the single-stranded region of the substrate, does not depends on the length of the single-stranded region and does not require ATPase or helicase activities. Notably, resection of the 3' overhang is precisely dependent on the presence of *bona-fide* telomeric repeat sequences in both the double and single-stranded regions of the DNA molecule, as any modification within the substrate that alters the TTAGGG repeat unit results in the complete inhibition of DNA processing. Importantly, sequence-specific processing of the telomeric substrate is inhibited by the single-stranded telomeric DNA binding protein POT1, suggesting a functional interplay between this protein and WRN in the homeostasis of the telomeric 3' overhang.

## Results

### Limited processing of telomeric DNA substrates with a 3' G-rich overhang by WRN exonuclease in vitro

**Figure 1.**
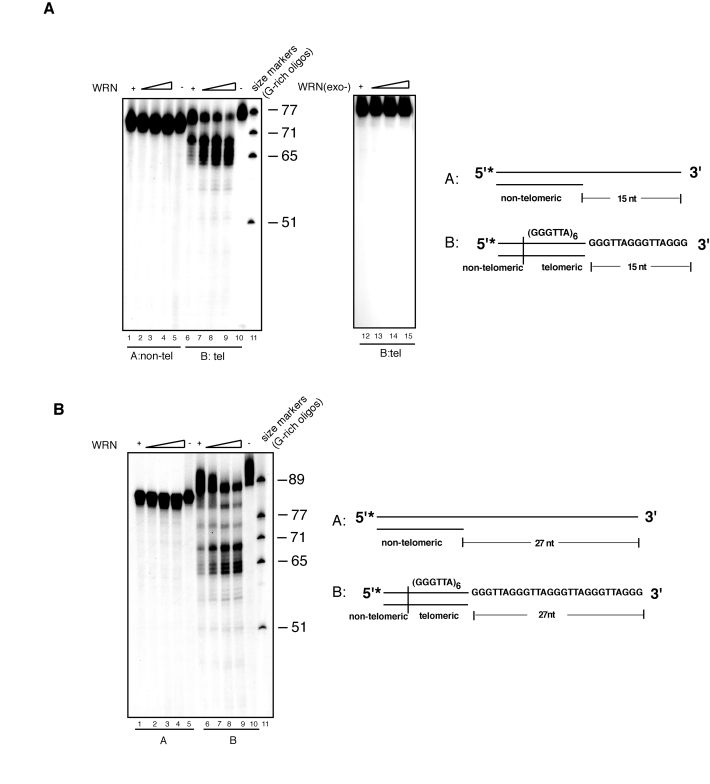
WRN exonuclease resects the 3' single-stranded overhang of telomeric DNA substrates. (**A**) 100 to 400 fmol of purified recombinant wild-type WRN or exonuclease mutant WRN (WRN D82A) were incubated with 5'-^32^P-labeled, 15 nt 3'-overhang DNA substrates containing non-telomeric sequences (*lanes 1-5*) or telomeric (TTAGGG) repeats (*lanes 6-10*) at 37°C for 10 min. The reaction products were analyzed by 12% polyacrylamide-urea denaturing gel and autoradiography (*lane 1 to 4*, 100, 200, 300, and 400 fmol of WRN; *lane 5*, non-telomeric DNA substrate; *lane 6 to 9*, 100, 200, 300, and 400 fmol of WRN; *lane 10*, telomeric DNA substrate; *lane 11, *(TTAGGG) repeats molecular size markers, *lane 12 to 15*, 100, 200, 300, and 400 fmol of exonuclease mutant WRN(D82A). (**B**) 100 to 400 fmol of purified recombinant WRN were incubated with 5'-^32^P-labeled, non-telomeric (*lanes 1-5*) or telomeric (*lanes 6-10*) DNA substrates with 27 nt 3'-overhang at 37°C for 10 min. The reaction products were analyzed by 12% polyacrylamide-urea denaturing gel and autoradiography (*lane 1 to 4*; 100, 200, 300, and 400 fmol of WRN; *lane 5*, non-telomeric DNA substrate; *lane 6 to 9,* 100, 200, 300, and 400 fmol of WRN; *lane 10, *telomeric DNA substrate*, lane 11*, (TTAGGG) repeats molecular size markers.

In previous studies, we and others have demonstrated that standard double-stranded DNA substrates with a 3' overhang are not significantly processed by WRN exonuclease *in vitro *[12,13,38,39]. Yet, the presence of WRN at telomeres prompted us to test whether telomeric sequences with a 3' single-stranded overhang are particularly susceptible to WRN-mediated processing. For this purpose, non-telomeric or telomeric DNA substrates with a 15 nucleotides 3' overhang were incubated with increasing amounts of purified WRN in cell-free exonuclease assays. DNA products from these reactions were separated by denaturing polyacrylamide gel electrophoresis and visualized by autoradiography. In agreement with our prior data [38], a non-telomeric double-stranded DNA substrate with a 3' overhang is not degraded by WRN (Figure 1). In contrast, we observed that WRN exonuclease removes several nucleotides from most of the 3' overhangs of the telomeric substrate. Notably, 3' processing of the telomeric substrate is not highly processive, as exonuclease activity slows down as it moves across the GGG trinucleotide repeats and in proximity of the junction between single-stranded and  double-stranded DNA, and produces weaker degradation further inward (Figure 1 and Supplementary Figure 1). Thus, the majority of the processing occurs within the single-stranded region of the DNA substrate leading to the generation of products with a shorter 3' overhang. This striking profile of DNA proces- sing is strictly dependent on a functional WRN, since degradation of the 3' overhang from the telomeric substrate is abolished when DNA is incubated with a mutant WRN lacking exonuclease activity (WRN-D82A).

To determine whether exonucleolytic processing was influenced by overhang length, we performed exonuclease assays using a telomeric substrate with a 27 nucleotides 3' overhang. The result of this experiment shows that this substrate is processed by WRN exonuclease to generate a periodic pattern of DNA products with shorter 3' overhangs (Figure 1B), while under the same experimental conditions incubation of WRN with a non-telomeric DNA substrate results in the removal of mostly one to three nucleotides from some of the substrate molecules without any significant furtherinward degradation (Figure 1B). This result is in agreement with a previous study, which reported limited degradation of substrates bearing a 3' overhang longer than 25 nucleotides by WRN [17]; (see also Supplementary Figure 1). Telomeric substrates with shorter overhangs but not substrates with non-telomeric sequences were also processed by WRN exonuclease (data not shown). Collectively, these results demonstrate that the exonuclease activity of WRN specifically processes telomeric DNA substrates with 3' overhang independently of the length of the single-stranded overhang.

### Concentration and time dependency of exonuclease activity on telomeric DNA substrate

**Figure 2.**
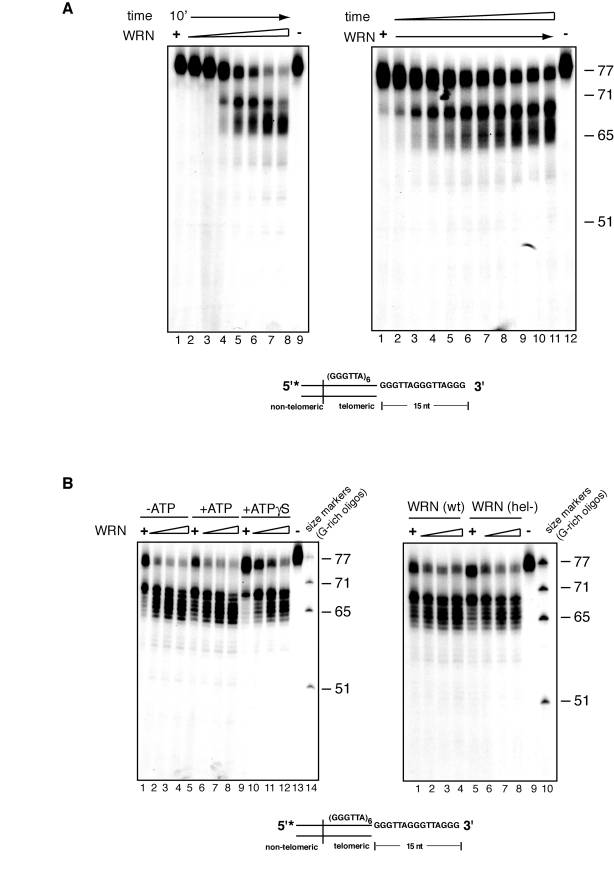
Concentration and time dependency of WRN exonuclease activity on telomeric substrates. (**A**) (*left*) 25 to 500 fmol of purified WRN were incubated with  5'-^32^P-labeled, 3'-overhang telomeric DNA substrate at 37°C for 10 min. The reaction products were analyzed by 12% polyacrylamide-urea denaturing gel and autoradiography (lane 1 to 10, 25, 50, 100, 150, 200, 250, 300, 350, 400, 500 fmol of purified WRN; lane 11, DNA substrate.  (*Right*) 200 fmol of purified WRN was incubated with 5'-^32^P-labeled, 3'-overhang telomeric DNA substrate at 37°C from 0 to 10 min. The reaction products were analyzed by 12% polyacrylamide-urea denaturing gel and autoradiography (lane 1 to 11, 0, 1, 2, 3, 4, 5, 6, 7, 8, 9 and 10 min; lane 12, DNA substrate. (**B**) (*left*) 100 to 400 fmol of purified WRN were incubated with 5' ^32^P-labeled, 3'-overhang telomeric DNA substrate in the absence or presence of 1.0 mM ATP or 1.0 mM Adenosine 5'-[γ-thio]triphosphate (ATPγS) at 37°C for 10 min. The reaction products were resolved by 12% polyacrylamide-urea denaturing gel and visualized by autoradiography (lane 1 to 4, 100, 200, 300, and 400 fmol of WRN without ATP; lane 5 to 8, 100, 200, 300, and 400 fmol of WRN in the presence of ATP, lane 9 to 12, 100, 200, 300, and 400 fmol of WRN in the presence of 1.0 mM ATPγS; lane 13, DNA substrate; lane 14, (TTAGGG) repeats molecular size markers. (*Right*) 100 to 400 fmol of purified WRN or WRN helicase mutant (K577M) were incubated with telomeric DNA substrates at 37°C for 10 min. The reaction products were analyzed by 12% polyacrylamide-urea denaturing gel and autoradiography (lane 1 to 4, 100, 200, 300, and 400 fmol of WRN; lane 5 to 8, 100, 200, 300, and 400 fmol of helicase mutant WRN; lane 9, DNA substrate; lane 10, (TTAGGG) repeats molecular size markers.

To examine in more details the kinetics of WRN-mediated processing of telomeric substrates with a 3' overhang, we titrated the amount of WRN added to the exonuclease reactions. As shown in Figure 2A, at low concentrations, WRN hydrolyzes a few nucleotides from the 3' overhang of a small percentage of telomeric substrate molecules, while increasing concentrations of enzyme results in the step-wise processing of increasing amounts of telomeric DNA substrate into a number of products with shorter 3' overhangs (Figure 2A). In a complementary experiment, the telomeric DNA substrate was incubated with WRN for 1 to 10 minutes and the products of the reactions were analyzed by denaturing polyacrylamide gel electrophoresis. The results of this experiment show that removal of a few nucleotides from the 3' overhang occurs within one minute of WRN addition and then continues inward to produce time-dependent accumulation of progressively smaller products differing by 1 to 6 nucleotides (Figure 2A).

### ATPase and helicase activities are not required for 3' processing of telomeric substrates

In addition to a 3' to 5' exonuclease activity, WRN possesses intrinsic ATPase and ATP-dependent helicase activities. To determine if these activities assist in the nucleolytic processing of the G-rich overhang of telomeric substrates, exonuclease reactions were carried out in the presence or absence of ATP, or the non-hydrolysable ATP analogue ATPγS. A similar pattern of DNA products is observed in the presence or absence of ATP, demonstrating that 3' end processing of telomeric substrates is independent of ATP hydrolysis (Figure 2C). Reactions carried out in the presence of ATPγS display a modest decrease in the amount of processed molecules, although the pattern of products is unaffected.

To confirm that ATPase and helicase activities do not influence the rate of extent of nucleolytic processing of the 3' overhang, a telomeric DNA substrate was incubated with a WRN variant lacking both activities (WRN K577M) and the products of the reactions were analyzed by denaturing polyacrylamide gel electro-phoresis.  As shown on the right panel of Figure 2B, the pattern and intensity of DNA products generated by WRN K577M, does not significantly differ from that of wild-type WRN. Identical results were obtained when WRN K577M was incubated with the telomeric substrate in the presence or absence of ATP (data not shown).

### Degradation of telomeric substrate by WRN is dependent on the presence of telomeric sequences in both the single and double stranded region of the DNA

**Figure 3.**
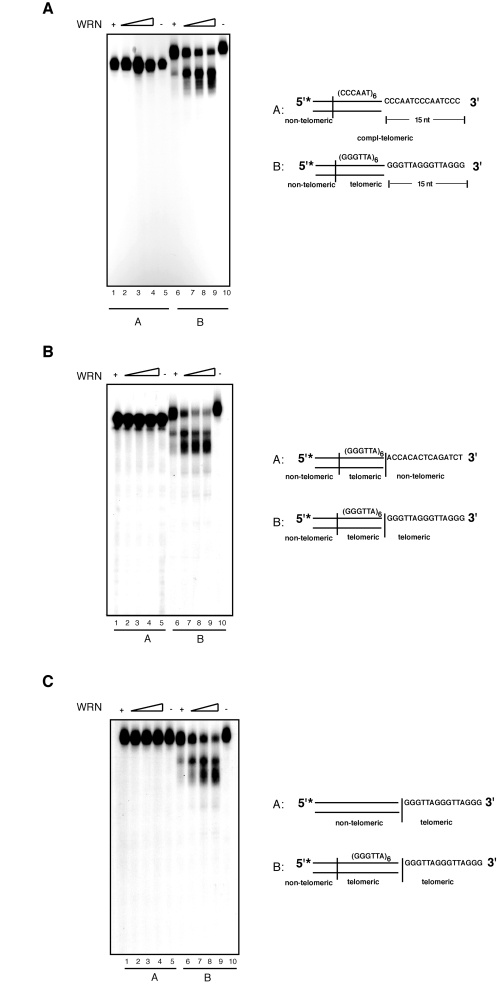
Both single- and double-stranded telomeric DNA sequences are required for the processing of the 3' overhang by WRN exonuclease. (**A**) 100 to 400 fmol of purified WRN were incubated with 5'-^32^P-labeled, 3'-overhang DNA substrate with  (CCCAAT) repeats sequence (*lanes 1-5*) and telomeric DNA substrate (*lanes 6-10*) at 37°C for 10 min. The reaction products were analyzed by 12% polyacrylamide-urea denaturing gel and autoradiography (*lane 1 to 4*, 100, 200, 300, and 400 fmol of WRN; *lane 5*, (CCCAAT) repeat DNA substrate; *lane 6 to 9*, 100, 200, 300, and 400 fmol of WRN; *lane 10*, telomeric DNA substrate.  (**B**) 100 to 400 fmol of purified WRN were incubated with 5'-^32^P-labeled DNA substrate with 3' of non-telomeric overhang (*lanes 1-5*) and telomeric DNA substrate (*lanes 6-10*) at 37°C for 10 min. The reaction products were analyzed by 12% polyacrylamide-urea denaturing gel and autoradiography (*lane 1 to 4*, 100, 200, 300, and 400 fmol of WRN; *lane 5*, telomeric DNA substrate with non-telomeric overnhang; *lane 6 to 9*, 100, 200, 300, and 400 fmol of WRN; *lane 10*, telomeric DNA substrate. (**C**) 100 to 400 fmol of purified WRN were incubated with 5'-^32^P-labeled, 3'-overhang DNA substrate with double-stranded non-telomeric sequence (*lanes 1-5*) and telomeric DNA substrate (*lanes 6-10*) at 37°C for 10 min. The reaction products were analyzed by 12% polyacrylamide-urea denaturing gel and autoradiography (*lane 1 to 4*, 100, 200, 300, and 400 fmol of WRN; *lane 5*, DNA substrate with double-stranded non-telomeric DNA sequence; *lane 6 to 9*, 100, 200, 300, and 400 fmol of WRN; *lane 10*, telomeric DNA substrate.

**Figure 4.**
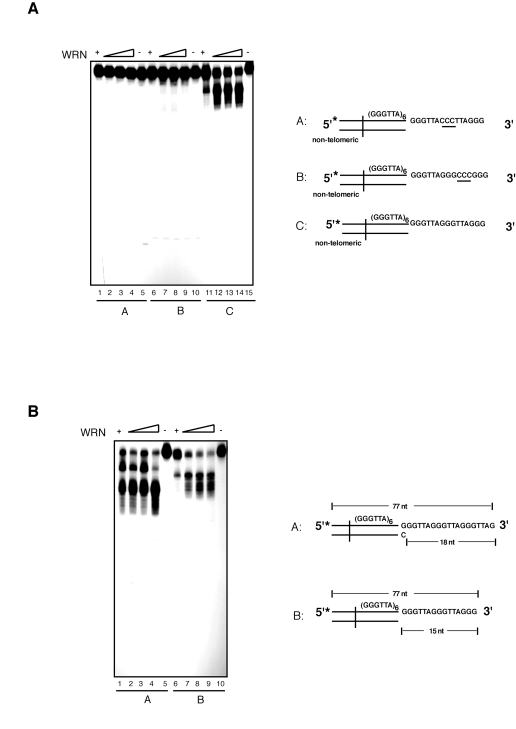
WRN exonuclease does not process telomeric DNA substrates with nucleotide substitutions within the 3' overhang sequence that alter the telomeric repeat unit. (**A**) 100 to 400 fmol of purified WRN were incubated with 5'-^32^P-labeled telomeric DNA substrates with nucleotide substitutions within the 3' overhang at 37°C for 10 min. The reaction products were analyzed by 12% polyacrylamide-urea denaturing gel and autoradiography (*lane 1 to 4*, 100, 200, 300, and 400 fmol of WRN; *lane 5*, telomeric DNA substrate with 3' overhang CCC (69 to 71) substitution (substrate A); *lane 6 to 9*, 100, 200, 200, and 400 fmol of WRN; *lane 10*, telomeric DNA substrate with 3'overhang CCC (72 to 74) substitution (substrate B); *lane 11 to 14*, 100, 200, 300, and 400 fmol of WRN; *lane 15*, telomeric DNA substrate C. (**B**) 100 to 400 fmol of purified WRN were incubated with 5'-^32^P-labeled telomeric DNA substrates with either 18 nt (substrate A) or 15 nt (substrate B) 3' overhangs at 37°C for 10 min. The reaction products were analyzed by 12% polyacrylamide-urea denaturing gel and autoradiography (*lane 1 to 4*, 100, 200, 300, and 400 fmol of WRN; *lane 5*, DNA substrate A; *lane 6 to 9*, 100, 200, 300, and 400 fmol of WRN; *lane 10*, DNA substrate B.

Our data indicates that the exonuclease activity of WRN on substrates with 3' overhangs is telomere-specific. To confirm the specificity of this reaction for 5'-TTAGGG-3' repeated sequences we carried out exonuclease assays with modified substrates. First, to determine whether the presence of a repeated motif was sufficient for 3' end processing by WRN, we tested a DNA substrate composed of several 5'-AATCCC-3' repeats with a 15 nucleotides C-rich 3' overhang (Figure 3A; compl-telomere). Incubation of this substrate with WRN does not yield any detectable processing product, even at the highest WRN concentration tested. Next, to determine if limited processing of the 3'overhang requires the presence of telomeric repeats in both the duplex portion of the DNA and 3' single-stranded overhang, WRN was incubated with substrates bearing altered sequences in either the double-stranded or single-stranded DNA region of the telomeric substrate. Changing the double-stranded portion of the telomeric substrate to non-telomeric sequences results in the complete inhibition of WRN exonuclease activity (Figure 3B).  Similarly, replacing the single-stranded 3' overhang from a telomeric repeat sequence to a non-telomeric sequence is sufficient to prevent DNA degradation by  WRN  (Figure 3C).  To corroborate the  strict requirement for the presence of precise telomeric 5'-TTAGGG-3' repeat units, we introduced three nucleotides substitutions at two distinct positions within the sequence of the G-rich 3' overhang. We measured the extent of WRN exonuclease activity on telomeric substrates in which the GGG triplet at nucleotides 69 to 71 or the TTA triplet at nucleotides 69 to 71 were replaced with a CCC triplet. The results of these experiments show that each of these changes within the 3' G-rich overhang is sufficient to completely inhibit WRN exonuclease activity on telomeric DNA substrates (Figure 4A). Collectively, these data demonstrate that WRN exonuclease activity on 3' overhangs of telomeric DNA substrates is strictly dependent on the presence of perfect telomeric TTAGGG motifs in both double-stranded and single-stranded regions of the substrate.

Next, to determine whether the identity of the 3' terminal nucleotide influences resection of the 3' end, we measured the extent of WRN exonuclease activity on a telomeric DNA substrate with a 5'-GGTTAG-3' terminal sequence. This is the preferred 3' terminal sequence of human telomeres *in vivo* [40]. As shown in Figure 4B, incubation of WRN with the telomeric DNA substrate terminating with the GGTTAG-3' sequence leads to a distinct pattern of products than the telomeric substrate terminating with the 5'-TTAGGG-3' sequence. Significantly, comparison of the digestion patterns indicates that resection of the 3' overhang of each substrate follows a sequence-specific pattern that stalls within the GGG trinucleotide unit (see also Supplementary Figure 1). These results indicate that the terminal nucleotide of the 3' overhang influences the pattern of products but does not alter exonuclease activity, further stressing the strict telomere sequence-specificity of the processing reaction.

### POT1 but not TRF2 inhibits 3' end processing of telomeric DNA substrates by WRN exonuclease

**Figure 5.**
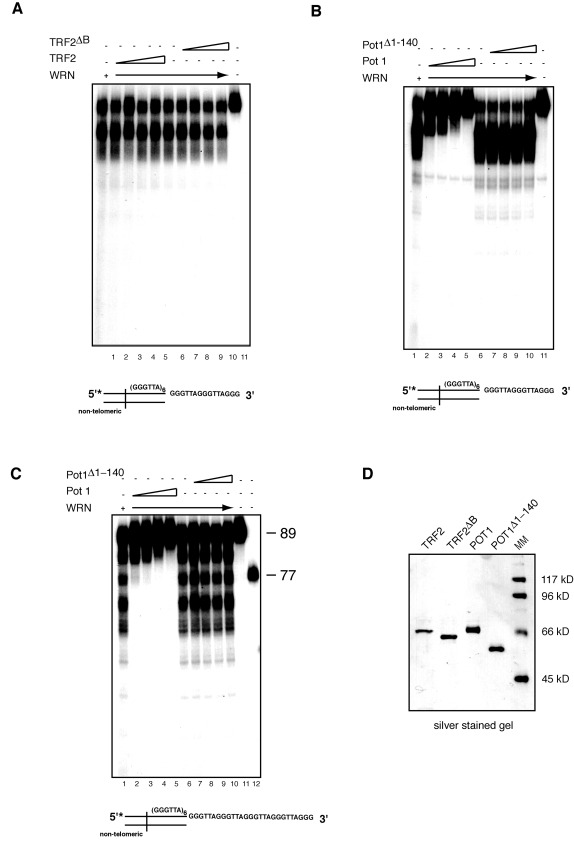
POT1 but not TRF2 inhibits processing of the 3' overhang of telomeric DNA substrates by WRN exonuclease. (**A**) 200 to 800 fmol of purified TRF2 or TRF2^Δ^^B^ were incubated with telomeric DNA substrates at room temperature for 20 min, then 200 fmol of WRN was added into the reaction to incubate at 37ºC for 15 min. The products were analyzed by 12% polyacrylamide-urea denaturing gel and autoradiography (*lane 1, *200 fmol of WRN*; lane 2 to 5*, 200, 400, 600, 800 fmol of TRF2 and 200 fmol of WRN; *lane 6, *200 fmol of WRN*; lane 7 to 10*, 200, 400, 600, 800 fmol of TRF2^Δ^^B^ and 200fmol of WRN; *lane 11*, DNA substrate. (**B**)200 to 800 fmol of purified POT1 or POT1^Δ^^1-140^ were incubated with telomeric DNA substrates at room temperature for 20 min, then 200 fmol of WRN was added into the reaction to incubate at 37ºC for 15 min. The products were analyzed by 12% polyacrylamide-urea denaturing gel and autoradiography (*lane 1, *200 fmol of WRN;* lane 2 to 5*, 200, 400, 600, 800 fmol POT1 and 200 fmol of WRN;* lane 6, *200 fmol of WRN; *lane 7 to 10*, 200, 400, 600, 800 fmol POT1^Δ^^1-140^ and 200 fmol of WRN; *lane 11*, DNA substrate). (**C**) 200 to 800 fmol of purified POT1 or POT1^Δ^^1-140^ were incubated with telomeric substrates with 27 nt 3' overhang at room temperature for 20 min, then 200 fmol of WRN was added into the reaction to incubate at 37ºC for 15 min. The products were analyzed by 12% polyacrylamide-urea denaturing gel and autoradiography (*lane 1, *200 fmol of WRN*; lane 2 to 5*, 200, 400, 600, 800 fmol of POT1 and 200 fmol of WRN; *lane 6, *200 fmol of WRN;*lane 7 to 10*, 200, 400, 600, 800 fmol of POT1^Δ^^140^ and 200 fmol of WRN; *lane 11*, 89-mer telomeric oligo, lane 12 77-mer telomeric oligo. (**D**) Silver stained SDS-PAGE gel showing the purity of purified recombinant TRF2, TRF2^Δ^^B^, Pot1, and POT1^Δ^^1-140^ used in the exonuclease assays.

TRF2 and POT1 are two components of the shelterin complex that function in the protection of telomeric ends [41]. TRF2 binds at the junction of the double/single stranded telomeric DNA sequence and interacts with WRN through its basic amino terminal domain [24]. To assess whether TRF2 modulates WRN exonuclease activity on telomeric DNA substrates, TRF2 or TRF2^Δ^^B^, a TRF2 mutant that does not bind WRN, were first preincubated with the telomeric DNA substrate at room temperature for 20 minutes, then WRN was added to the reaction and the incubation was continued at 37^0^C for an additional 15 minutes. The results of this experiment, which are shown in Figure 5A, indicate that neither TRF2 nor TRF2^Δ^^B^ influence the pattern of digested products, demonstrating that TRF2 does not alter the pattern of DNA processing of telomeric overhangs by WRN.

Next we tested whether POT1 affects WRN exonuclease activity. The single-stranded telomeric DNA binding protein POT1 plays a key role in telomere end protection and telomere length regulation [41]. POT1 has been shown to stimulate WRN helicase activity on forked telomeric substrates, however no effect on WRN exonuclease was reported, at least in the context of the substrates used in this study [22]. To determine whether POT1 influences the processing of the 3' G-rich single-stranded overhang by WRN, we preincubated POT1 or a mutant form of POT1 (POT1^Δ^^1-140^), which lacks the single-stranded DNA binding domain, with a telomeric DNA substrate at room temperature for 20 minutes before the addition of WRN and further incubation at 37^0^C for 15 minutes. Gel shift assays with a radiolabeled (GGGATT)_5_ single-stranded telomeric oligonucleotide confirmed that POT1 but not POT1^Δ^^1-140^ binds to single-stranded telomeric DNA (data not shown). The results of the exonuclease assays indicate that addition of increasing amounts of POT1 inhibits the processing of substrates with either a 15 or 27 nucleotides 3' single-stranded telomeric overhang by WRN exonuclease (Figure 5B and C).  Importantly, the DNA binding activity of POT1 is required to prevent the limited processing of the 3' telomeric overhang by WRN, since POT1^Δ^^1-140^ is unable to repress WRN exonuclease activity on both substrates (Figure 5B and C).

## Discussion

In this study we investigated the biochemical properties of the WRN protein at telomeres and describe *in vitro* studies that expose a unique property of WRN exonuclease on 3' single-stranded overhang of telomeric DNA substrates mimicking natural telomere termini. Here, we demonstrate that WRN exhibits sequence-specific exonuclease activity that specifically removes, in a restricted fashion, several nucleotides inward from the G-rich 3' single-stranded overhang of telomeric substrates. Close inspection and comparison of the digestion pattern among the different substrates used in this study (see Supplementary Figure 1) reveals that processing of each telomeric substrate occurs readily up to the first GGG repeat unit where it stalls; it then proceeds further inward until the next GGG repeat where it slows down again, and this pattern continues until WRN approaches the double-stranded single-stranded junction where further digestion is dramatically reduced. This activity requires the presence of telomeric sequences in both the duplex and 3' G-rich overhang DNA segments, as replacements in either the double-stranded portion or the single-stranded DNA extension that alter the normal GGGATT telomeric repeat unit result in the complete inhibition of WRN exonuclease.

Several groups have characterized the activity of WRN exonuclease on 3' recessed duplex DNA substrates or blunt duplexes with an internal bubble or fork on the opposite end [12,14,16,42,43]. However, only one group has previously reported that WRN exonuclease can digest 3' overhangs of sufficient length [17]. This study reported that WRN degrades non-telomeric DNA substrates with more than 20 nucleotides overhangs to generate products that are generally one nucleotides shorter [17]. Interestingly, while WRN ATPase and helicase activities do not influence processing of telomeric substrates (Figure 2B), WRN exonuclease on non-telomeric 3' overhang substrates is stimulated by ATP hydrolysis [17].  These results demonstrate that the ATPase and helicase activities play a role in the processing of 3' overhangs of non-telomeric but not telomeric substrates, and suggest that WRN can resect 3' overhangs through two distinct mechanisms, one of which is telomeric repeats sequence specific. As we observed a minor but reproducible inhibition of DNA processing by ATPγS, it is possible that nucleotide binding without hydrolysis may induce a conformational change in WRN that limits the attack of telomeric substrates.

WRN is a nuclear protein that has been implicated in several nuclear processes ranging from DNA repair, transcription, recombination and telomere metabolism [18].  This multiplicity of cellular functions is reflected in the variable pattern of localization of this protein in the nucleus [44,45]. Significantly, immuno-histochemical studies and chromatin immuno-precipitation assays have revealed that a subpopulation of WRN is associated with chromosome ends, primarily during the S-phase of the cell cycle [19], and lack of WRN or expression of an helicase-negative WRN protein in normal cells causes telomere loss [19,46]. *In vitro* studies have further indicated that WRN cooperates with telomeric repeat binding factors TRF1 and TRF2 to unwind the telomeric displacement loop (D-loop), a structure formed by invasion of the 3' single-stranded telomeric overhang of internal homologous double-stranded DNA region [20]. Although these studies have exposed an important function for the WRN helicase activity in the maintenance of telomere homeostasis, our understanding of how WRN regulates telomere length and stability is far from being well understood. Moreover, the role that the exonuclease activity plays in this and possibly other processes remains unknown. Unraveling the cellular function of WRN exonuclease activity is of critical importance, since this is the domain that radically differentiates WRN from other human RecQ helicases such as BLM and RecQ4 [47].

Nucleolytic activities at telomeres must be properly controlled, since failure to restrain it would cause telomere attrition and chromosome instability, which may affect cell viability by inducing cell senescence or apoptosis, or promote tumorigenesis [37,41,48]. To prevent unwanted degradation, telomeres are bound by telomere-specific factors known as the shelterin complex that collectively contribute to the protection of telomere termini from excessive processing or fusion with other telomere ends. Significantly, we found that the telomeric protein POT1 but not TRF2 inhibits WRN processing of the 3' overhang, suggesting that POT1, in addition to its role in protecting the 5' telomeric end from nucleolytic resection, is important for regulating WRN-mediated processing of the 3' telomeric overhang. A previous study has reported that POT1 influence WRN helicase activity, however no alterations in exonuclease activity by POT1 were observed [22].  It is likely that lack of inhibition of WRN exonuclease activity by POT1 was due to the nature of the telomeric substrate used in this study, which was a forked blunt-ended DNA duplex.

Our demonstration that WRN exhibits sequence-specific exonuclease activity that specifically removes several nucleotide inward, in a controlled manner, from the G-rich 3' overhang of telomeric substrates, suggests that WRN exonuclease activity may play a role in telomere homeostasis. We envision that WRN exonuclease activity at chromosome ends may have an important role in configuring the telomere 3' termini prior to the formation of the protective t-loop structure and shaping the ends of telomeres for proper replication, telomerase elongation, or protection. Future studies testing the function of WRN exonuclease activity at chromosome ends *in vivo* will be critical for defining the role of WRN and its activities in telomere homeostasis.

## Methods

DNA substrates used in this study:

1. 77-mer (G77/C62) telomeric substrate with 15 nucleotides 3' overhang

5'AGCTGAGCATGTCCAGACATGTCCTAGGGTTAGGGTTAGGGTTAGGGTTAGGGTTAGGGTTAGGGTTAGGGTTAGGG3' 5'TCGACTCCTACAGGTCTGTACAGGATCCCAATCCCAATCCCAATCCCAATCCCAATCCCAAT3'

2. 77-mer (77/62) non-telomeric substrate with 15 nucleotides 3'overhang

5'AGCTGAGCATGTCCAGACATGTCCTACCAGAGCCATATGAGTCAAACCGTCATCGAGCTCCGTGTGAACTAGCTCATTCGAC3' 5'TCGACTCGTACAGGTCTGTACAGGATGGTCTCGGTATACTCAGTTTGGCAGTAGCTCGAGGC3'

3. 77-mer non-telomeric substrate with telomeric 3' overhang sequence

5'AGCTGAGCATGTCCAGACATGTCCTACCAGAAGCCATATGAGTCAAACCGTCATCGAGCTCCGGGGTTAGGGTTAGGG3' 5'TCGACTCGTACAGGTCTGTACAGGATGGTCTTCGGTATACTCAGTTTGGCAGTAGCTCGAGGC3'

4. 77-mer telomeric substrate with non-telomeric 3'overhang sequence

5'AGCTGAGCATGTCCAGACATGTCCTAGGGTTAGGGTTAGGGTTAGGGTTAGGGTTAGGGTTAACCACACTCAGATCT3' 5'TCGACTCGTACAGGTCTGTACAGGATCCCAATCCCAATCCCAATCCCAATCCCAATCCCAAT3'

5. 89-mer (G89/C62) telomeric substrate with 27 nucleotides 3'overhang

5'GGGTTAGGGTTAAGCTGAGCATGTCCAGACATGTCCTAGGGTTAGGGTTAGGGTTAGGGTTAGGGTTAGGGTT AGGGTTAGGGTTAGGG3' 5'TCGACTCCTACAGGTCTGTACAGGATCCCAATCCCAATCCCAATCCCAATCCCAATCCCAAT3'

6. 89-mer (89/62) non-telomeric substrate with 27 nucleotides 3'overhang

5'ACCTGCAACTAGAGCTGAGCATGTCCAGACATGTCCTACCAGAGCCATATGAGTCAAACCGTCATCGAGCTCCG TGTGAACTAGCTCAT3' 5'TCGACTCGTACAGGTCTGTACAGGATGGTCTCGGTATACTCAGTTTGGCAGTAGCTCGAGGC3'

7. 77-mer telomeric substrate with mutant (69-CCC) 3' overhang

5'AGCTGAGCATGTCCAGACATGTCCTAGGGTTAGGGTTAGGGTTAGGGTTAGGGTTAGGGTTAGGGTTACCCTTAGGG3' 5'TCGACTCCTACAGGTCTGTACAGGATCCCAATCCCAATCCCAATCCCAATCCCAATCCCAAT3'

8. 77-mer telomeric substrate with mutant (72-CCC) 3' overhang

5'AGCTGAGCATGTCCAGACATGTCCTAGGGTTAGGGTTAGGGTTAGGGTTAGGGTTAGGGTTAGGGTTAGGGCCCGGG3' 5'TCGACTCCTACAGGTCTGTACAGGATCCCAATCCCAATCCCAATCCCAATCCCAATCCCAAT3'

9. 77-mer (CCCTAA) repeat sequence substrate with 3'overhang (compl-telomeric)

5'AGCTGAGCATGTCCAGACATGTCCTACCCAATCCCAATCCCAATCCCAATCCCAATCCCAATCCCAATCCCAATCCC3' 5'TCGACTCCTACAGGTCTGTACAGGATGGGTTAGGGTTAGGGTTAGGGTTAGGGTTAGGGTTA3'

10. 77-mer Telomeric substrate with 18 nucleotides 3' overhang and terminal GTTAG sequence

5'AGCTGAGCATGTCCAGACATGTCGGGTTAGGGTTAGGGTTAGGGTTAGGGTTAGGGTTAGGGTTAGGGTTAGGGTTA3' 5'TCGACTCCTACAGGTCTGTACAGCCCAATCCCAATCCCAATCCCAATCCCAATCCCAAT3'

11. 70mer non-telomeric substrate with 25 nucleotides 3' overhang

5'GCTGATCAACCCTACATGTGTAGGTAACCCTAACCCTAACCCTAAGGACAACCCTAGTGAAGCTTGTAAC3' 5'CGACTAGTTGGGATGTACACATCCATTGGGATTGGGATTGGGATT3'

All telomeric DNA substrates have a 26 nucleotides non-telomeric 5' end to allow proper annealing of the oligonucleotides.

Protein expression and purification. Recombinant Flag-tagged wild-type WRN, exonuclease mutant WRN (D82A), helicase mutant WRN (K577M), TRF2, TRF2^Δ^^B^, POT1, and POT1(Δ1-140) cDNAs were cloned into baculovirus expression vectors to generate recombinant viruses used to infect Sf9 cells. 48 hours after infection, cells were collected and lysed in lysis buffer (10 mM Hepes pH 7.5, 100 mM NaCl, 1.5 mM MgCl2, 0.5% Nonidet P-40). Recombinant proteins were purified by DEAE-cellulose and affinity chromatography on anti-Flag resin [38-39].

Exonuclease assay. DNA exonuclease activity of was measured as described in [39]. Briefly, oligonucleotides were labeled at the 5' end with [^32^P] ATP and T4 polynucleotide kinase. The appropriate oligonucleotides were then annealed by boiling followed by slowly cooling to room temperature. Reaction mixtures containing 50 fmol of DNA substrate (100,000 cpm) and increasing amounts of WRN in 40 mM Tris-HCl pH 7.5, 4 mM MgCl2, 5 mM dithiothreitol, 0.1mg/ml bovine serum albumin, in the absence or presence of 1 mM ATP or 1 mM ATPγS, in a final volume of 10 μl were incubated at 37ºC for 10 minutes. Reactions were terminated by the addition of 2.0 μl of a 95% formamide solution and after incubation at 95°C for 3 minutes, DNA products were resolved by 12% polyacrylamide-urea gel electrophoresis and visualized by autoradiography.

To assess WRN exonuclease activity in the presence of TRF2 and POT1 variants, telomeric DNA substrates (50 fmol, 100,000 cpm) were incubated with increasing amounts (200-800 fmol) of either POT1, POT1(Δ1-140), TRF2 or TRF2^Δ^^B^ in 10 μl of Buffer A (10 mM Tris-HCl pH 7.5, 80 mM NaCl, 4 mM KCl, 4 mM MgCl2, 1mM ATP, and 5% glycerol) at room temperature for 20 minutes. Then, 200 fmol of WRN was added into the reaction mixture and incubate at 37ºC for an additional 15 minutes. Each reaction was then terminated by the addition of 2.0 μl of a 95% formamide solution. After incubation at 95 °C for 3 minutes, DNA products were resolved by 12% polyacrylamide-urea gel electrophoresis and visualized by autoradiography.

## Supplementary figure

Supplementary Figure 1 Processing of telomeric substrates with 3' overhangs by WRN exonuclease. (**A**) 100 to 400 fmol of purified WRN were incubated with 5'-radiolabeled non-telomeric DNA substrate with 25 nt 3' overhang or telomeric substrate with 15 nt 3' overhang at 37°C for 10 min. The reaction products were analyzed by 12% polyacrylamide-urea denaturing gel and autoradiography (*lane 1 to 4*, 100, 200, 300, and 400 fmol of WRN; *lane 5*, nont-telomeric DNA substrate; *lane 6 to 9*, 100, 200, 300, and 400 fmol of WRN; *lane 10*, telomeric DNA substrate; *lane 11*, G-rich molecular size markers. (**B**) *(left)* 400 fmol of purified recombinant wild-type WRN was incubated with 5'-^32^P-labeled 3'-overhang telomeric DNA substrates at 37°C for 10 min. The reaction products were resolved on a long 12% polyacrylamide-urea denaturing gel to improve bands resolution and visualized by autoradiography. M= G-rich telomeric oligonucleotides were used as molecular size markers. (*right*) Schematic representation of substrates used in the exonuclease assay. Arrows and brackets denote major processing products identified in this study.
