# Supplementary figures and images for: Sequence-specific processing of telomeric 3' overhangs by the Werner syndrome protein exonuclease activity
Source: Aging (Albany NY). 2009 Mar 17;1(3):289–302. doi: 10.18632/aging.100032 (PMC2806009; doi:10.18632/aging.100032)

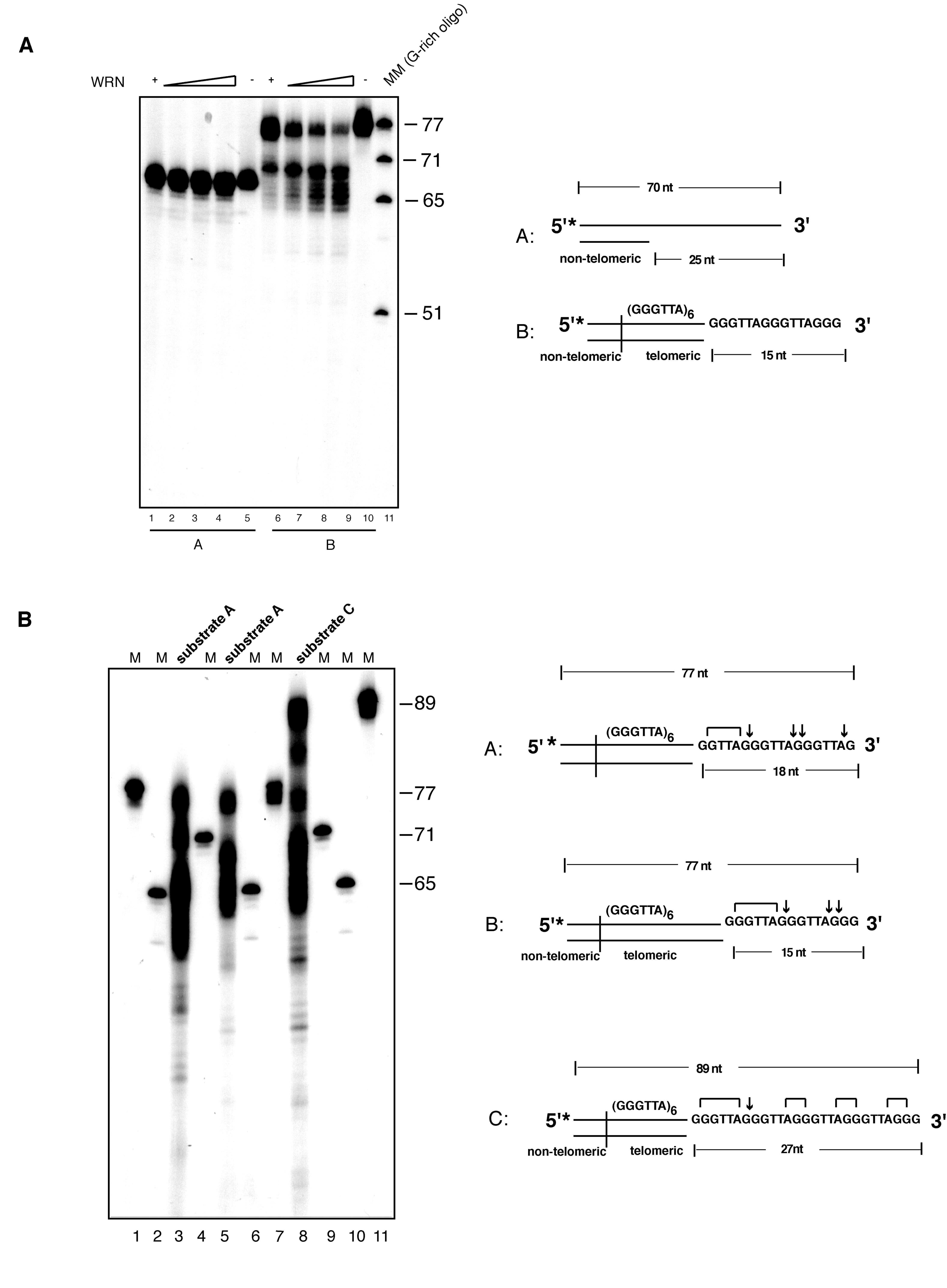

Supplement: Supplementary Figure 1 — (A) 100 to 400 fmol of purified WRN were incubated with 5'-radiolabeled non-telomeric DNA substrate with 25 nt 3' overhang or telomeric substrate with 15 nt 3' overhang at 37°C for 10 min. The reaction products were analyzed by 12% polyacrylamide-urea denaturing gel and autoradiography (lane 1 to 4, 100, 200, 300, and 400 fmol of WRN; lane 5, nont-telomeric DNA substrate; lane 6 to 9, 100, 200, 300, and 400 fmol of WRN; lane 10, telomeric DNA substrate; lane 11, G-rich molecular size markers. (B) (left) 400 fmol of purified recombinant wild-type WRN was incubated with 5'-32P-labeled 3'-overhang telomeric DNA substrates at 37°C for 10 min. The reaction products were resolved on a long 12% polyacrylamide-urea denaturing gel to improve bands resolution and visualized by autoradiography. M= G-rich telomeric oligonucleotides were used as molecular size markers. (right) Schematic representation of substrates used in the exonuclease assay. Arrows and brackets denote major processing products identified in this study. [file aging-01-289-s001.tif]
